# Joint spectro-temporal and perceptual feature learning using a dual-track attention network for music genre classification

**DOI:** 10.3389/frai.2026.1824319

**Published:** 2026-06-25

**Authors:** V. Adithya Hari, S. Alden Jenish, R. Karthik, M. Ananya

**Affiliations:** 1 School of Electronics Engineering, Vellore Institute of Technology, Chennai, India; 2 Centre for Cyber-Physical Systems Vellore Institute of Technology, Chennai, India

**Keywords:** attention mechanism, constant-Q transform (CQT), deep learning, dual-track architecture, music genre classification

## Abstract

**Objectives:**

Music Genre Classification (MGC) plays a pivotal role in information retrieval, underpinning the organization, recommendation, and discovery of music. Current approaches predominantly depend on spectrogram-based convolutional models or handcrafted acoustic features, which inadequately capture the intricate interplay between spectral, temporal, and perceptual cues that define musical genres.

**Methods:**

To overcome these limitations, we introduce a dual-track architecture that fuses local spectro-temporal textures with global statistical descriptors through an attention-driven framework. The first track utilizes the Efficient Axis Fusion Residual Attention Network (EAFRAN), incorporating Hybrid Attention Residual Fusion (HARF) and Axis-Integrated Contextual Attention (AICA) modules to model complex time-frequency relationships. The second track employs the Residual Convolutional-Attention Embedding Network (RCAEN), leveraging streaming low-level descriptors and residual multi-head self-attention layers to encode global perceptual features. The complementary feature maps from both tracks are combined to yield an efficient and discriminative representation of musical content.

**Results:**

Experiments conducted on the GTZAN benchmark dataset demonstrated that the proposed model attained an accuracy of 98.47 ± 0.6%, outperforming existing state-of-the-art methods.

**Conclusion:**

The dual-track attention-based strategy effectively bridges local and global musical features, achieving efficient classification performance and offering a potential foundation for enhanced music information retrieval systems.

## 1 Introduction

Music is an organized arrangement of sounds over time and is expressed by vocals or instruments. It is composed of the essential elements such as melody, harmony, rhythm, and timbre. These elements, when combined, define and distinguish one style of music from another. Music incorporates an aesthetic aspect while also representing social and cultural characteristics and self-expression. Digital music repositories have emerged, providing access to large libraries and changing how people search for, discover, and engage with them (Tzanetakis and Cook, 2002). Music genres are a vital factor in this ecosystem, which helps organize and access music content efficiently. Music streaming services facilitate music classification and discovery using intuitive genre labels, which helps users navigate vast libraries and receive personalized music recommendations based on their music preferences and histories (Karim et al., 2019). Hence, Music Genre Classification (MGC) has become an important field of research to automate the classification of audio recordings by genre without human intervention.

Conventionally, MGC has relied on handcrafted features, created to highlight particular aspects of the audio. These features may range from magnitude-based, pitch-based, chord-based, and timbre-based, such as Mel-Frequency Cepstral Coefficients (MFCC) and Root Mean Square (RMS) energy features. However, these features often lacked robustness across datasets because they struggled to capture the subtle spectro-temporal changes across genres that might differentiate them. Hence, spectrograms have become more notable in MGC, as they provide a joint time–frequency representation of audio and many other signals. In parallel, intelligent audio analysis systems have accelerated the migration towards data-driven models like Machine Learning (ML) and Deep Learning (DL), which are able to learn discriminative representations from large datasets. These methods remove the need for manual feature engineering by hierarchically extracting important features directly from raw or minimally processed data. Thus, ML and DL models are playing a critical role in the advancement of automatic genre classification by providing reliable pattern recognition, adaptive generalization, and enhanced interpretability to complex and heterogeneous musical data. A reliable and robust MGC is required to provide improved music recommendations and user experience in modern streaming services. This research aims to leverage a DL-based framework for MGC by incorporating spectrogram-based Convolutional Neural Network (CNN) encoders with low-level feature embeddings.

## 2 Related works

Elbir and Aydin (2020) utilized the Mel spectrogram of each segment divided from the music track. The study presented a custom CNN architecture for MGC and recommendation. Ghildiyal et al. (2020) also employed Mel spectrograms generated from audio samples for MGC. The study sliced the generated spectrograms into 64 strips, thereby increasing the sample size, and utilized a custom CNN model for feature extraction and classification. Xu (2024) presented a comparative analysis of different CNN and Recurrent Neural Network (RNN) models for MGC. Costa et al. (2016) implemented a deep CNN model along with a Support Vector Machine (SVM) for MGC, leveraging both spectral and handcrafted features. The audio signals were converted to spectrograms using the Hann window with discrete Fourier transform, followed by Mel and Bark scale zoning to capture visual and acoustic textures. Nanni et al. (2015) employed an ensemble-based framework that integrates both acoustic and visual audio representations. The study performed feature selection for dimensionality reduction and presented final classification through ensemble fusion of SVM, random subspace, and AdaBoost classifiers using a voting mechanism. Tzanetakis and Cook (2002) introduced an audio analysis network using Short-Term Fourier Transform (STFT)-based windowing for detailed temporal-spectral resolution. The study employed multi-pitch detection and circle-of-fifths mapping to capture pitch-related attributes and a Gaussian mixture model and k-NN as classifiers. You et al. (2024) presented self-supervised contrastive learning using log-Mel spectrograms and a custom CNN model for open-set audio and genre classification. Salazar (2023) introduced Completed Local Binary Pattern (CLBP)-based multipartite complex network representations for MGC. The study converted audio signals into spectrograms, from which CLBP codes were computed and used to construct monopartite, bipartite, and tripartite complex networks. Topological and textural features extracted from these networks are concatenated and classified using traditional ML algorithms.

Narkhede et al. (2021) presented a comparative analysis of different ML algorithms for MGC from audio-based low-level descriptors. Yu et al. (2019) employed deep attention-based bidirectional RNNs to refine temporal feature weighting from STFT spectrograms for MGC. Ashraf et al. (2023) presented hybrid CNN-RNN variants to compare the effectiveness of Mel-spectrograms and MFCC representations for MGC. Cheng et al. (2020) also utilized Mel-spectrograms generated by fast Fourier transform coupled with majority voting-based custom CNN for MGC. Liu et al. (2020) employed a bottom-up broadcast neural network with stacked inception modules and dense connections to achieve improved performance and parameter efficiency. The study utilized STFT-based spectrograms and log-scaling for perceptual uniformity. Kumaraswamy and Poonacha (2021) presented a deep CNN for MGC. The study employed a self-adaptive sea lion optimization algorithm to tune model parameters and feature weights for improved performance. Tian (2025) utilized a hybrid deep neural network combining the fractional grey lag goose optimization algorithm for fine-tuning model parameters, enabling efficient exploration of search space and improved performance. Jenifer et al. (2025) implemented both waveform and spectrogram representations, capturing complementary time and frequency domain information. The time-domain features and spectral centroid-based representations were transformed into 2D images and processed using a lightweight CNN model. Li (2024) presented a dual parallel CNN architecture incorporating a black hole optimization algorithm for hyperparameter tuning. The study converted audio signals to mono before extracting MFCC and STFT-based spectral features, followed by Z-score normalization for consistency. Zhang and Li (2025) employed a parallel CNN ensemble strategy with a capuchin search-driven tuning algorithm for efficient MGC. The audio signals were converted to mono, and features were extracted using discrete wavelet transform, MFCC, and STFT. Zhang et al. (2025) utilized a pretrained Zeiler and Fergus network to extract high-level features from audio signals and extreme learning machines for MGC. The study also employed a modified metaheuristic algorithm to optimize model parameters for improved performance. Dentamaro et al. (2025) implemented a ResNet-50 CNN trained on logarithmically scaled Mel-spectrograms for MGC.

In summary, this section presents existing MGC approaches from shallow, feature-engineered approaches to sophisticated deep learning models enhanced by hybridization, optimization, and multimodal fusion. However, there still exist limitations, such as the sensitivity of subtle spectral features and ambiguous genre boundaries, that hinder the development of efficient systems.

### 2.1 Research gaps

This research aims to present solutions to the existing gap and challenges in music genre classification. The research gaps include:

1. Existing methods often depend solely on either representations from spectrograms or handcrafted statistical feature descriptors. Spectrograms effectively encapsulate short-term spectral and temporal dynamics but do not characterize intricate, more global semantic or descriptive information. While handcrafted statistical features summarize the audio content more globally, they typically do not provide the necessary representational capability for effective generalization across diverse datasets.
2. Conventional CNN architectures have been effective in learning local spectro-temporal patterns. However, they have limited ability to model higher-order dependencies and long-range temporal relations in complex musical pieces.
3. Fewer studies have explored the integration of heterogeneous feature modalities as a unified model. It limits the model’s ability to learn complementary feature representations when spectral and statistical descriptors are considered individually. The absence of integration limits the ability to leverage the set of discriminative information available to musical signals.

### 2.2 Contributions

The proposed work presents novel contributions to mitigate the identified research gaps in MGC. The contributions include:

1. The study proposes a dual-track architecture that incorporates CQT spectrograms and low-level descriptors to classify music genres. By leveraging heterogeneous modalities, the model captures fine-grained spectro-temporal characteristics and global descriptive attributes to improve classification performance.
2. The EAFRAN utilizes CQT spectrograms along with tailored attention mechanisms to capture long-range contextual dependencies within the spectral domain. In parallel, the second track, RCAEN, processes low-level audio descriptors using residual attention building blocks to enhance global context modeling and hierarchical feature abstraction. The proposed system effectively learns cross-modal interactions and complementary feature learning, helping to preserve local-scale spectral textures and global temporal semantics for MGC efficacy.
3. The study combines the latent representations from both the tracks prior to the classification process. This strategy enables the model to capture synergistic interactions between spectral and statistical modalities, resulting in improved discriminative capability and performance.

## 3 Proposed network

**Figure 1:**
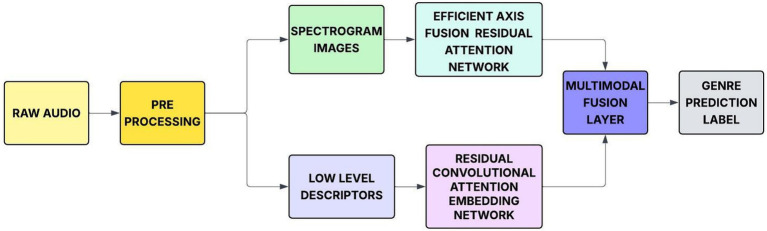
Schematic representation of the proposed network.

The proposed model utilizes a dual-track architecture that leverages CQT spectrograms for local and long-range structure and low-level acoustic descriptors to model global perceptual cues. The latent representations of both the tracks are combined to enable the model to learn temporal and global aspects, creating an efficient network for classification of music genres. A schematic representation of the proposed network is given in Figure 1.

### 3.1 Efficient axis fusion residual attention network (EAFRAN)

**Figure 2:**
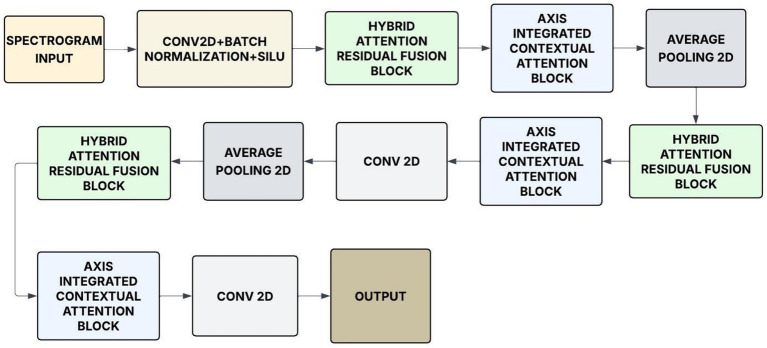
Schematic representation of the EAFRAN network.

The EAFRAN serves as the first track for spectro-temporal feature extraction in the proposed MGC. The track extracts hierarchical spectro-temporal features to model complex frequency and time-domain dependencies. The network introduces a progressive refinement hierarchy, denoting changes from raw spectrograms to contextual semantic representations through a multi-stage process of localized enhancements and global context integration. Initially, the EAFRAN utilizes a convolution module that transforms the input spectrograms into a low-level feature space. These feature maps are subsequently processed by hierarchical pairs of HARF and AICA blocks. The HARF blocks are designed to progressively refine the local spectral representations by emphasizing spectro-temporal relevance, ensuring feature consistency across successive hierarchical stages. Through selective activation and differentiable feature reuse, these blocks help preserve prominent harmonic, tonal, and rhythmic patterns across the network. Following the HARF block, an AICA block is employed to enable the model to capture cross-dimensional dependencies. The AICA blocks improve the model’s interactions between temporal dynamics and spectro-temporal variations and aid the modeling of both localized modulations and broader contextual relationships. This design helps the network learn fine-grained, context-aware embeddings effectively, thereby improving the discriminative capability for MGC. An overview of the EAFRAN is illustrated in Figure 2.

#### 3.1.1 Hybrid attention residual fusion (HARF) block

**Figure 3:**
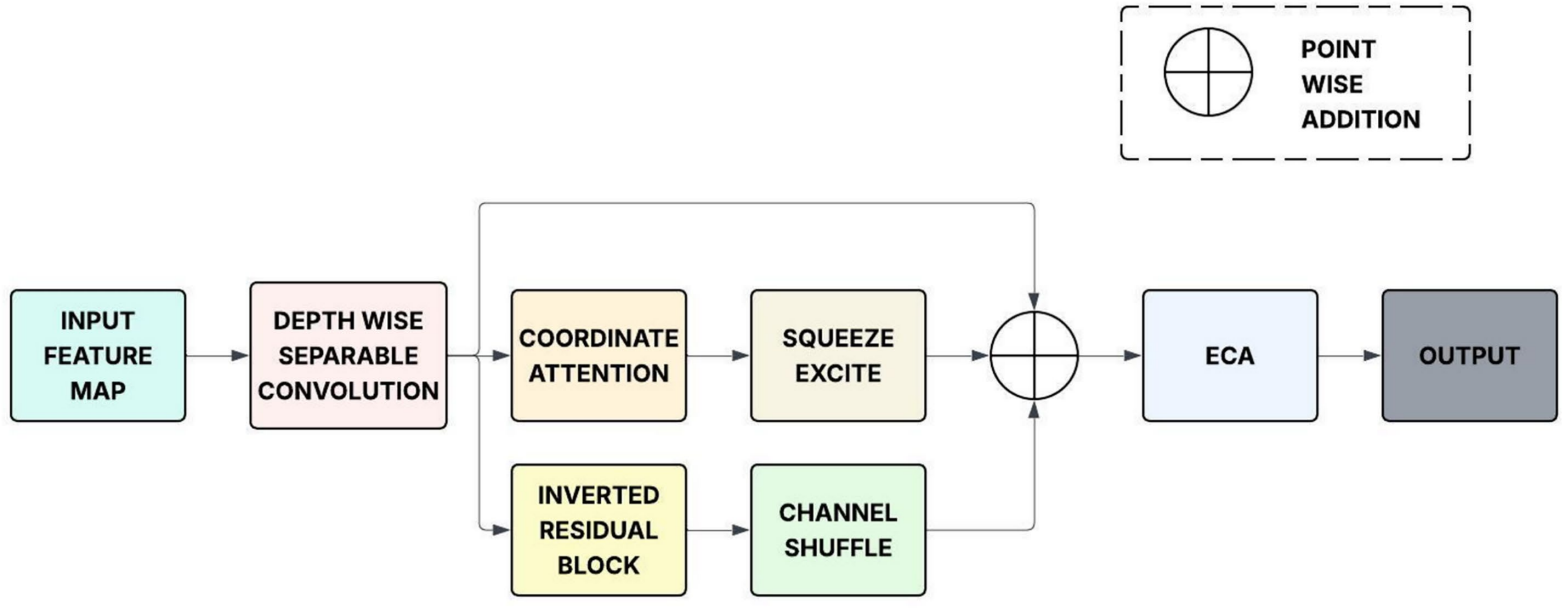
Schematic representation of the HARF block.

The HARF is used to refine intermediate spectro-temporal representations using hierarchical feature-aggregation and attention-enhancement procedures. This block helps between spectral locality preservation and global context efficiency, allowing extracted features to retain both localized fine harmonic detail and wider structural coherence. It decompose spectrograms into time- and frequency-specific subspaces, reducing computational cost while attending to fine timbral granularity (Chen et al., 2019). The convolutional feature maps are then filtered and enhanced using coordinate attention and squeeze-excitation to adaptively rescale and reweight channel-wise and spatial feature responses, enhancing discriminative cues (Hou et al., 2021; Hu et al., 2018). Furthermore, inverted residual learning and channel shuffle modules are employed in parallel to maintain representational efficiency (Zhang et al., 2018; Sandler et al., 2018). This enables the model to redistribute information across channel groups and reduce redundancy in feature representation. Before residual aggregation and point-wise addition, tensor dimensions from parallel branches are explicitly aligned to ensure spatial consistency. Since the coordinate attention, inverted residual, and contextual attention generate feature maps with different temporal or frequency resolutions after convolutional operations, bilinear interpolation is employed to resize intermediate tensors to a common spatial resolution. After alignment, the refined feature maps are combined through residual pointwise addition: This dimensional harmonization stabilizes hierarchical feature fusion and preserves structural continuity across spectro-temporal representations. The aggregated output is then processed using the Efficient Channel Attention (ECA) module that applies attention to informative channels based on inter-channel dependencies with minimal computational requirements (Wang et al., 2020). The ECA stabilizes gradient propagation and ensures attention to musically salient elements, such as second harmonic and rhythm contours. Figure 3 illustrates the structural overview of the HARF block.

#### 3.1.2 Axis-integrated contextual attention (AICA) block

**Figure 4:**
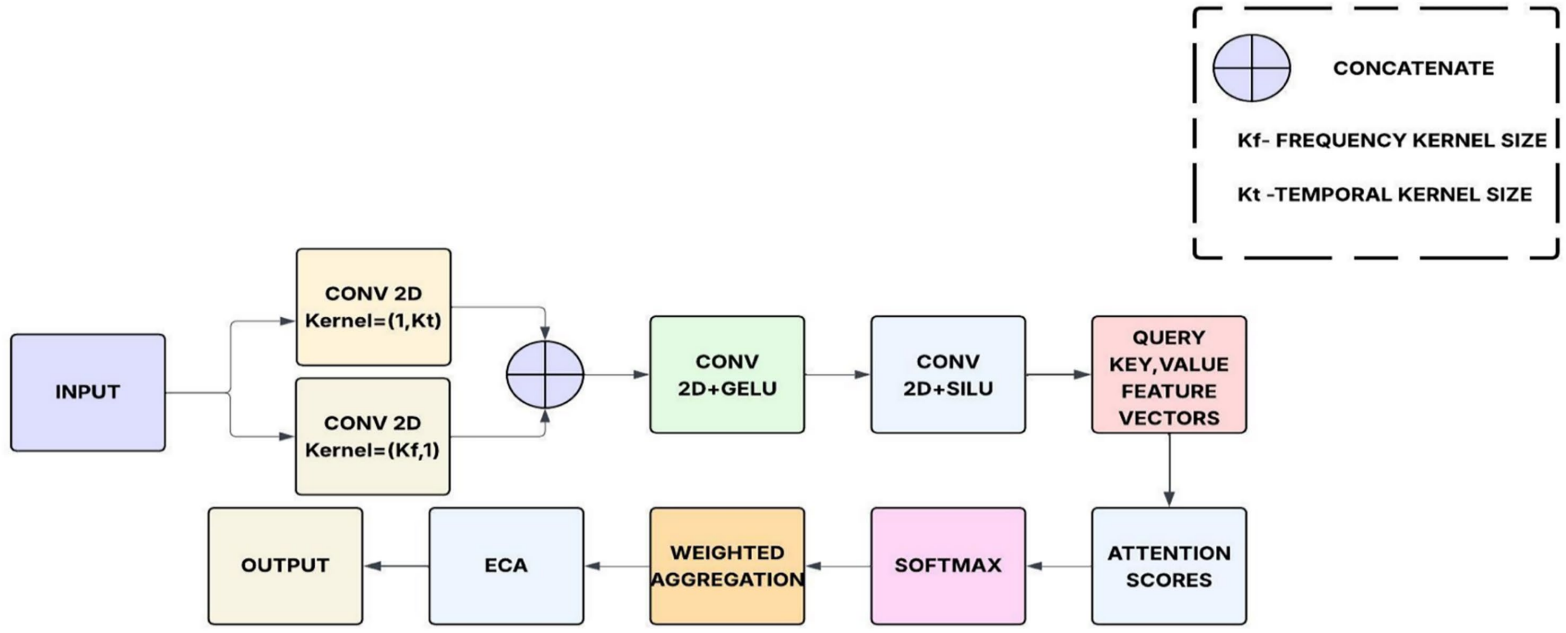
Schematic representation of the AICA block.

The AICA block acts as a feature interaction module in the EAFRAN framework, balancing the representation of musical spectrograms across temporal and frequency-domain dimensions. The AICA block allows contextual integration across axes, enabling the model to access frequency-time related features. Firstly, the directional dependencies along the frequency and time axes are captured using convolutional modules. Subsequently, long-range temporal relationships and discriminative sub-bands are captured, enhancing rhythmic and harmonic contexts along the length of the sequence. The contextual attention branch first compresses the frequency dimension using adaptive average pooling, generating a representation $X_{p}\in {\mathbb{R}}^{\text{BxCxT}}$ , where B, C, and T denote batch size, channels, and temporal length, respectively. A shared pointwise Conv1D projection maps $X_{p}$ to an intermediate tensor ${\mathbb{R}}^{\mathrm{Bx}3\mathrm{CxT}}$ , which is then partitioned into three equal tensors along the channel dimension to yield Query (Q), Key (K), and Value (V) matrices, as per Equation (1).


$$Q,K,V=\text{Split}(\text{Conv}1D(X_{p};X_{\mathrm{qkv}}))$$ (1)

where $X_{\mathrm{qkv}}$ denotes the 1×1 convolution weight of output size 3C, and the split is performed along the channel axis (dim = 1), yielding Q, K, V. The attention operation is then computed along the temporal dimension using scale dot product attention given in Equation 2.


$$A=\text{Softmax}(\frac{Q^{T}K}{\sqrt{C}})$$ (2)

where $A\in {\mathbb{R}}^{\text{BxTxT}}$ models temporal dependencies across T time steps. The output is subsequently obtained by weighting the V matrix with the attention map, as given in Equation 3.


$$O={\left(\mathrm{Ax}V^{T}\right)}^{T}$$ (3)

where $O\in {\mathbb{R}}^{\text{BxCxT}}$ is the post attention feature map that recovers the channel-temporal structure of the input. This output is subsequently interpolated back to the original spectrogram resolution before residual fusion. The ECA module is employed to adaptively weigh informative channels in the original audio feature set to support a reinforcement of relevant feature dimensions while stabilizing the propagation of features. The collaboration between these submodules provides balanced representations of contextual coding across a spatial, temporal, and channel-wise context that improves robustness and discrimination across a broader range of musical styles. Figure 4 illustrates the architectural overview of the AICA block.

### 3.2 Residual convolutional-attention embedding network

**Figure 5:**
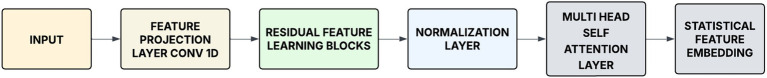
Schematic overview of the RCAEN.

The RCAEN functions as an additional processing track that utilizes low-level acoustic descriptors to capture global perceptual cues and statistical dependencies beyond the spectral domain. The RCAEN employs an initial convolutional projection to generate enhanced feature embeddings from descriptor vector representations, followed by a hierarchical stack of residual convolutional blocks while maintaining local continuity and attenuating information loss. For each segment, temporal, spectral, and perceptual descriptors, including RMS energy, spectral centroid, spectral bandwidth, spectral roll-off, spectral flatness, zero-crossing rate, chroma statistics, and MFCC coefficients, are computed. Statistical aggregation using mean and variance operations is subsequently performed for every descriptor sequence to obtain compact global representations. This enables the RCAEN track to model inter-descriptor dependencies using residual convolutions and multi-head self-attention operations. The residual connections allow stable gradient flow and leverage the network’s ability to model subtle inter-feature variations resulting from complex descriptor distributions. A multi-head self-attention mechanism is used to add global context into the representation, enabling the model to encode semantic relationships across descriptors. The final representation is compacted using adaptive pooling and a nonlinear projection, producing a discriminative embedding that encodes both fine-grained local information and overall relationships amongst descriptors to support the model’s ability to classify music genres with robust context. Figure 5 presents the schematic overview of the RCAEN track.

### 3.3 Classification

The final classification of the proposed architecture combines the complementary representations extracted from the EAFRAN and RCAEN tracks for efficient MGC. Initially, the output feature maps for the EAFRAN track are processed using global adaptive average pooling to obtain the spectro-temporal embedding. Similarly, the output features of RCAEN are processed to obtain the descriptor-aware contextual embedding. These representations are projected into a compatible latent feature space using learnable linear transformation layers prior to the fusion. This projection operation ensures dimensional consistency and enables balanced contribution from both modalities during feature fusion. The projected embeddings are subsequently merged through feature-wise concatenation. The concatenation-based fusion layer is learnable indirectly through the preceding projection layers and subsequent fully connected classification layers, allowing the network to adaptively optimize cross-modal feature interactions during training. This fusion strategy preserves modality-specific information while enabling joint contextual learning between spectro-temporal and descriptor-based representations. To further refine the fused representation and improve generalization, the combined embedding is passed through batch normalization and dropout regularization. The refined feature vector is then processed using a fully connected layer with ReLU activation to generate higher-level discriminative representations. Finally, a linear classification head maps the learned representation to the target genre categories, and the softmax activation function is applied during inference to obtain normalized class probabilities.

## 4 Experiments and results

This section presents the dataset utilized, preprocessing methods employed, and the environmental setup for the study. Subsequently, the section also provides information about the tuned hyperparameters, comparison with existing models and studies, evaluation with external datasets, and the ablation experiments conducted.

### 4.1 Dataset description and preprocessing

The study utilizes the GTZAN Genre Collection dataset, an established benchmark dataset for music genre classification tasks. The dataset, curated by Tzanetakis and Cook (2002), consists of 1,000 audio recordings categorized into 10 specific genres. It holds exactly 100 tracks per genre, and every audio sample is 30 s long, in mono 16-bit WAV format at the sampling rate of 22,050 Hz.

The CQT-based spectrograms are generated, offering a logarithmic frequency resolution aligned with human musical perception in the first stream. To enrich this spectral representation, a three-channel spectrogram is constructed by using the magnitude spectrum and the logarithmic amplitude conversion. The delta coefficients are used to preserve the harmonic components and the temporal spectral dynamics. In parallel, the second track focuses on low-level statistical feature extraction, computing temporal, spectral, and perceptual descriptors. These include features like RMS for temporal analysis; centroid, bandwidth, roll-off, and flatness for frequency characterization; and MFCCs for perceptual modeling. A sliding window is used with 8-s chunks of audio, with an overlap of 4 s, to augment the dataset while ensuring it has continuity. The train, validation, and test splits were performed at the original track level prior to chunk generation to ensure that all overlapping segments derived from a recording remained within the same split. Performance metrics are reported at the segment level.

### 4.2 Experimental setup

The experiments were performed on the Kaggle platform using an NVIDIA Tesla T4 GPU. The model was implemented using the PyTorch framework, and the Librosa library was used to generate CQT spectrograms. The optimizer AdamW was used with a learning rate of 1e-4 and a weight decay of 1e-4. The objective function was cross-entropy loss, combined with dropout and batch normalization, which were used to mitigate overfitting. The dataset was divided into 70% for training and 15% each for validation and testing samples for assessment, with accuracy, precision, recall, and F1 score used for performance metrics.

To ensure reproducibility and reduce the impact of stochastic variation during training, all experiments were repeated using four independent random seeds: 42, 123, 256, 512, and 1,024. These seed values were selected to provide diverse initialization conditions and were not chosen based on model performance. For each run, all random number generators in PyTorch, NumPy, and CUDA were initialized with the corresponding seed, and the model weights were reinitialized before training. The reported accuracy, precision, recall, and F1-score values correspond to the mean and standard deviation computed across all five runs.

### 4.3 Hyperparameter tuning

Hyperparameter tuning was employed to achieve improved model performance and stable training. This includes tuning four main aspects: (1) the learning rate, (2) dropout rate, (3) weight decay, and (4) optimizer. In addition, different learning rates, ranging from $10^{-3}$ to $10^{-5}$ in addition to dropout values, typically placed between 0.20 and 0.50, were evaluated. Different optimizers were tested, with AdamW documented to have improved convergence consistency and improved generalization. Table 1 presents the summary of the hyperparameter tuning.

**Table 1:** Summary of the tuned hyperparameters.

Hyperparameter	Values	Optimal Value
Learning rate	[ $10^{-3}$ , $10^{-5}$ ]	10^−4^
Dropout rate	[0.2, 0.5]	0.3
Weight decay	[ $10^{-3}$ , $10^{-5}$ ]	10^−4^
Optimizer	[SGD, Adam, AdamW]	AdamW
Loss function	[Cross-Entropy, Focal]	Cross Entropy Loss

### 4.4 Performance analysis with state-of-the-art networks

A comparative study was conducted to measure the performance of the proposed architecture compared to a number of standard DL architectures for music genre classification. The vision transformer among the baseline models achieved the lowest performance level, with a recall of 60.00%, precision of 60.23%, F1-score of 59.75%, and accuracy of 60.00%, indicating a limited capability to capture fine-grained audio features. More traditional CNNs, including AlexNet, VGG-16, SqueezeNet, ResNet, and DenseNet, provided moderate improvements. Recent architectures such as EfficientNet, XceptionNet, and Pyramid Vision Transformer provided additional improvements, with recall, precision, and accuracy ranging from 76 to 78.8%. These models demonstrate the capability of DL for representations and better efficiency of attention on channel-wise features. VGG-19 outperformed most baseline networks, achieving a recall of 90.08%, a precision of 90.29%, and an accuracy of 90.08%, which demonstrates its capability to learn hierarchical features. Comparatively, the proposed model presented improved performances, including a recall of 98.47 ± 0.6%, a precision of 98.5 ± 0.5%, an F1-score of 98.4 ± 0.5%, and an accuracy of 98.47 ± 0.6%, showing the utility of integrating CQT-based spectro-temporal features with low-level acoustic descriptors to provide a comprehensive representation. The comparison highlights the improved ability of the proposed model to simultaneously capture fine-grained spectro-temporal details, along with structured statistical cues, during robust classification of music genres. Table 2 presents the summary of the comparative analysis.

**Table 2:** Comparative analysis with state-of-the-art networks.

Models	Recall (in %)	Precision (in %)	F1-Score (in %)	Accuracy (in %)
ViT	60.00	60.23	59.75	60.00
ResNet-50	66.2	66.5	65.19	66.66
DenseNet121	70.00	71.65	69.33	70.00
AlexNet	72.50	72.33	72.22	72.50
VGG-16	72.87	74.4	72.99	72.84
SqueezeNet	73.00	72.70	72.67	73.00
ResNet-18	74.00	74.00	73.62	74.00
DeIT	74.50	73.58	73.37	74.50
Swin Transformer	75.50	76.83	75.79	75.50
Pyramid Vision Transformer	76.00	78.23	76.37	76.00
DenseNet169	76.67	77.00	80.18	76.67
EfficientNet	71.14	74.64	71.94	71
EfficientNetB1	77.50	77.52	77.19	77.50
Mobilenet_V1	78.00	78.48	77.80	78.00
EfficientNetB3	78.00	79.12	77.46	78.00
EfficientNetB4	78.50	79.52	78.09	78.50
XceptionNet	78.8	79.8	78.5	78.8
ResNet-34	79.50	78.73	80.06	79.50
VGG-19	90.08	90.29	90.10	90.08
Proposed Network	98.47 ± 0.6	98.5 ± 0.5	98.4 ± 0.5	98.47 ± 0.6

### 4.5 Comparison with existing studies

The section presents the comparative analysis of the proposed system with existing research works for MGC that utilized the GTZAN dataset. The CNN-based approaches achieved moderate performance, denoting the foundational capabilities of convolutional models in capturing spectro-temporal patterns from audio signals. Subsequently, the hybrid methods integrating CNN-RNN models achieved improved accuracies, highlighting the effectiveness of temporal context modeling. Additionally, advanced architectures and enhanced CNN architectures presented competitive metrics compared to the other models. In comparison, the proposed system outperformed the existing research studies by achieving an accuracy of 98.47 ± 0.6% in the GTZAN dataset. Table 3 presents the summary of the analysis.

**Table 3:** Comparative analysis with existing works.

S. No	Source	Method	Accuracy (in %)
1.	Cheng et al. (2020)	Custom CNN	84
2.	Kumaraswamy and Poonacha (2021)	Custom CNN	85.63
3.	Ashraf et al. (2023)	Hybrid CNN-RNN	89.3
4.	Ghildiyal et al. (2020)	Custom CNN	91
5.	Yu et al. (2019)	Bi-directional RNN	92.7
6.	Li (2024)	Custom CNN	95.2
7.	Elbir and Aydin (2020)	Custom CNN	97.6
8.	Proposed system	Dual-track Attention Network	98.47 ± 0.6

### 4.6 Performance evaluation with external datasets

This section presents the performance of the proposed architecture when evaluated on two external datasets to assess the model’s generalizability. The results highlight the efficacy and adaptability of the proposed network to unseen data, demonstrating its potential in different research and music information retrieval.

#### 4.6.1 Evaluation on the Arabic music dataset

The proposed architecture was evaluated on the publicly available Arabic music genre classification dataset (Tawil, 2022). The dataset is a comprehensive collection of 1,268 Arabic audio recordings designed for multiclass and multilabel classification tasks. The clips are categorized into five distinct musical genres and styles: Muwashahat, east, loyal, poems, and rai. The network’s performance was measured across these varied categories to test its ability to distinguish between complex rhythmic and melodic structures inherent in Arabic music. The proposed model achieved an accuracy of 96.98%, with precision, recall, and F1-score of 97.35, 95.69, and 96.44%, respectively. These results highlight the model’s generalization ability and its effectiveness in processing diverse linguistic and instrumental audio features within the Arabic music domain.

#### 4.6.2 Evaluation on the ballroom dataset

The study also evaluated the proposed network on the publicly available Ballroom dataset (Krebs et al., 2013). The dataset is a specialized collection of audio recordings designed for rhythmic analysis, specifically focusing on beat and bar annotation across various dance styles. It consists of 698 audio files and is categorized by metrical levels and tempo ranges derived from specific dance style labels. The proposed model achieved an accuracy of 96.34%, with precision, recall, and F1-score of 94.95%, 95.14%, and 95%, respectively. The obtained results demonstrate the model’s efficacy in tracking rhythmic patterns and temporal features within the ballroom music domain. Table 4 summarises the performance of the proposed system across the tested external datasets.

**Table 4:** Summary of performance metrics obtained by the proposed network on external datasets.

Source	Dataset	Method	Accuracy (in %)
Medhat et al. (2017)	Ballroom	Masked Conditional Neural Network	90.4
Pons et al. (2016)	Ballroom	CNN	92.10
Peeters (2011)	Ballroom	SVM	96.13
Almazaydeh et al. (2022)	Arabic Music	VGG	95.00
Almazaydeh et al. (2022)	Arabic Music	AlexNet	96.00
Proposed system	Ballroom	Dual-track Attention Network	96.34
Proposed system	Arabic Music	Dual-track Attention Network	96.98

### 4.7 Ablation studies

This section presents a set of ablation studies that were performed to assess the independent and combined effects of the important elements of the proposed dual-track architecture for MGC. The analysis was executed using (1) the EAFRAN track that processes spectrograms to assess its ability to learn local and global spectro-temporal patterns; (2) the RCAEN track, which processes low-level acoustic descriptors to learn global perceptual cues and structural correlations; and (3) the dual-track architecture, which merges both the spectral and statistical methods through joint embedding fusion to investigate the complementary aspects of both modalities. Each track was assessed quantitatively using standard performance metrics such as accuracy, precision, recall, and F1-score.

#### 4.7.1 Analysis of the EAFRAN network

This section evaluates the performance of the EAFRAN, which relies on CQT-based spectrogram representations to encode sophisticated spectro-temporal dependencies for music genre classification. The EAFRAN specializes in extracting feature representations by leveraging stacked convolutional layers that capture harmonic textures, timbral cues, and rhythmic variations. The EAFRAN achieved an accuracy of 97.81%, a precision of 97.82%, a recall of 97.75%, and an F1-score of 97.76% on the test set. The results validate that a synergistic interaction between hierarchical refinement and adaptive axis attention substantially enhances spectro-temporal feature learning, thereby optimizing genre classification performance.

#### 4.7.2 Analysis of the RCAEN network

This section evaluates the performance of the RCAEN in the proposed dual-track architecture. The RCAEN utilizes low-level audio descriptors that include the spectral, temporal, and perceptual dimensions of the audio signal. The descriptors are derived as mean and variance in fixed-length segments to capture both central tendencies and sound variety associated with them. These representations complement features drawn from spectrograms to improve the discriminative capability of the system. On the test set, the RCAEN track achieved an accuracy of 81.90% and a precision, recall, and F1-score of 82.63%, 82.09%, and 81.97%, respectively. The results demonstrate the RCAEN track performed effectively to model audio patterns and contribute to the overall performance of the proposed system.

#### 4.7.3 Analysis of the proposed network

**Figure 6:**
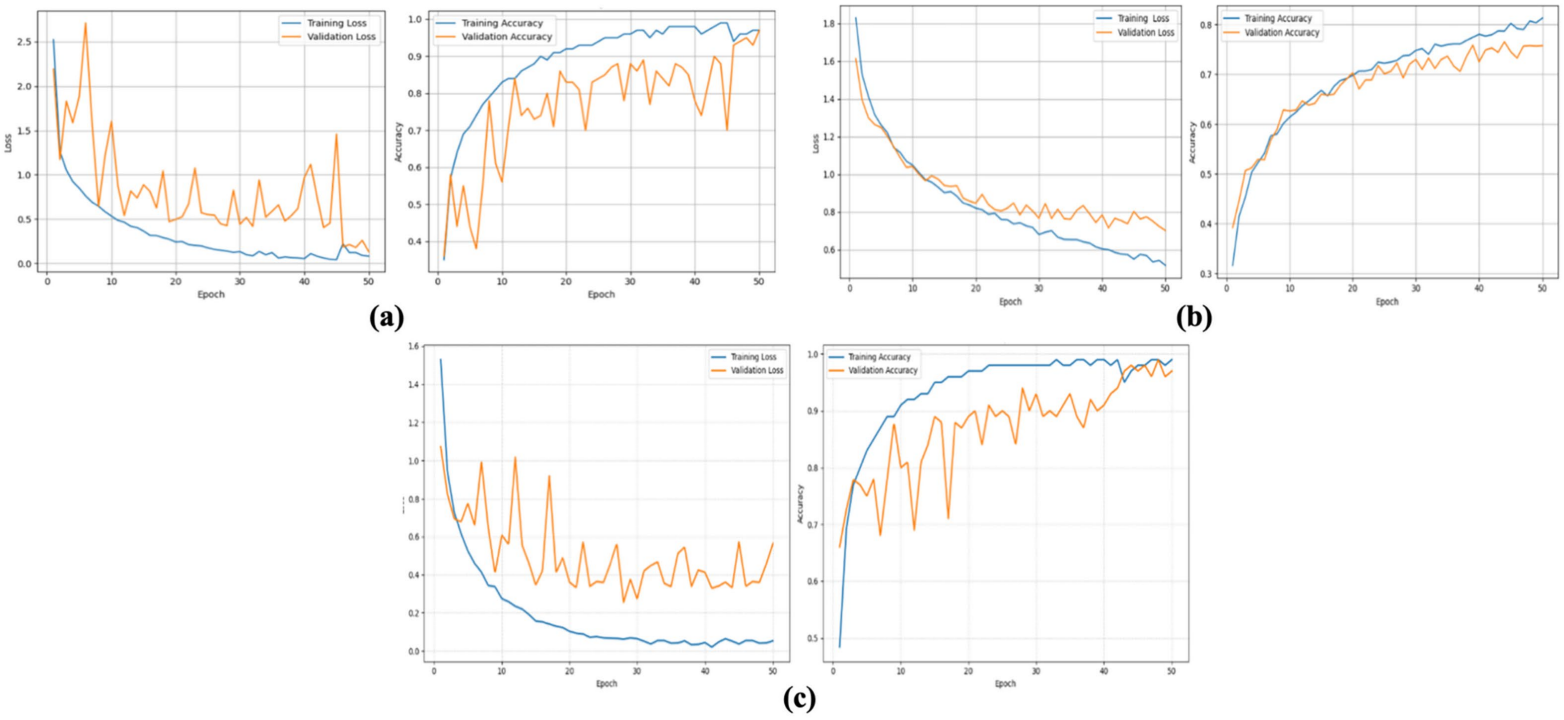
Loss and accuracy graphs of the ablation studies conducted: **(a)** EAFRAN, **(b)** RCAEN, and **(c)** Proposed Network.

**Figure 7:**
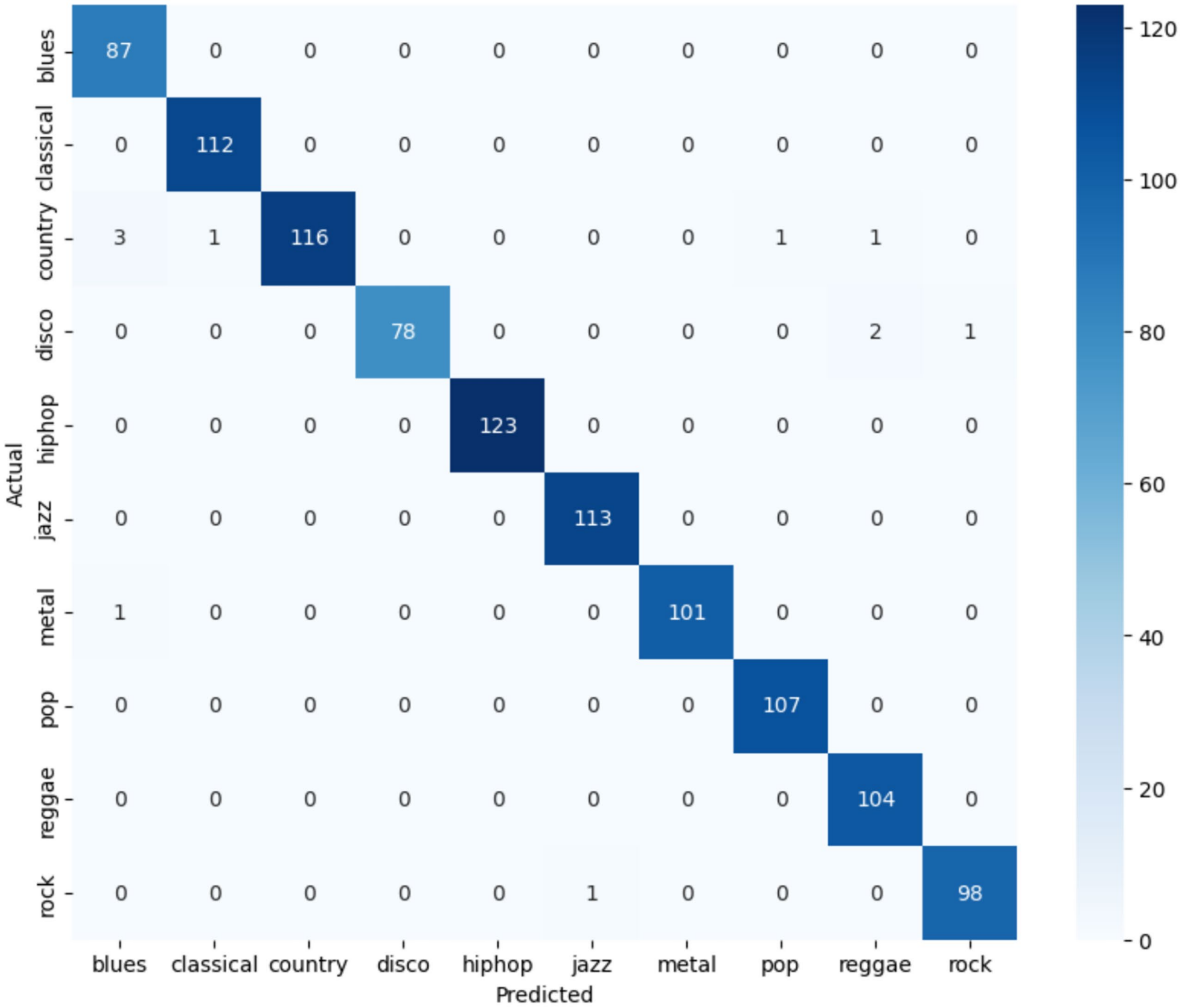
Confusion matrix for the proposed model.

This section presents the evaluation results of the proposed architecture. The model consists of EAFRAN, which utilizes spectrograms, and RCAEN, which leverages low-level descriptors. The tracks present complementary feature representations of the audio, aiding with efficient classification performance. The EAFRAN track utilizes hierarchical convolutional processing to capture local spectro-temporal patterns in the audio into discriminative deep features, while RCAEN encodes statistical descriptors using residual and attention mechanisms. The mean and variance of these local descriptors allows RCAEN to capture behavior and variations in the signal. The outputs of EAFRAN and RCAEN are concatenated into a single embedding, which is pooled and classified into genre labels via a classification head. By jointly modelling local spectro-temporal features and structured statistical features, the proposed architecture achieves efficient discrimination among genres. The observed training and validations graphs of the ablation studies are illustrated in Figure 6. To access the efficacy and reproducibility of the proposed architecture, additional experiments were conducted using five random seed values. The best-performing individual run achieved an accuracy of 99.14%, precision of 99.16%, recall of 99.14%, and F1-score of 99.14%. The final reported performance was computed as the mean and standard deviation across all five runs. The proposed network achieved an average accuracy of 98.47 ± 0.6%, precision of 98.5 ± 0.5%, recall of 98.47 ± 0.6%, and F1-score of 98.4 ± 0.5%, demonstrating both strong classification performance and consistent behavior across different random initializations. Table 5 presents the summary of the ablation studies conducted. Figure 7 presents the confusion matrix of the proposed network.

**Table 5:** Summary of ablation studies.

Experiment	Accuracy (in %)	Precision (in %)	F1-Score (in %)	Recall (in %)
EAFRAN	97 ± 0.3	97 ± 0.2	97 ± 0.3	97 ± 0.3
RCAEN	81 ± 0.5	81 ± 0.4	81 ± 0.4	81 ± 0.4
Proposed network	98.47 ± 0.6	98.5 ± 0.5	98.4 ± 0.5	98.47 ± 0.6

## 5 Limitations and future works

This section discusses the potential limitations identified in the current study and the future scope of research in MGC.

- The GTZAN dataset used in this study has limited environmental and scale diversity. The GTZAN dataset’s audio samples have been recorded under controlled and balanced conditions but do not represent the conditions present during a live performance, including various sources of background noise and multiple instruments overloading. Future studies should utilize large and heterogeneous datasets that can help enhance robustness and generalization for MGC in diverse acoustic scenarios.
- The proposed system primarily focuses only on the audio modality for the MGC. This may limit its contextual understanding of genre semantics. Since many music genres are influenced significantly by instrumentation, lyrics, cultural cues, and mood, the intricate semantics may not be sufficiently modeled solely by acoustic signals. Future work could expand the classifications that are produced through the system by incorporating multimodal data fusion approaches that include the use of text, symbols, and metadata to enhance the quality and visibility of the classifications produced by the system.
- The proposed system primarily works on genre-level classification and does not consider the possibility for genre variability or multi-label overlaps. This assumption of musically exclusive labels may limit the model’s ability to predict real-world libraries, where a single music sample may exhibit characteristics of multiple genres. Upcoming research should consider extending the MGC frameworks to include multi-label genre tagging, hierarchical classification, or style transfer learning. This enables effective and fine-grained recognition of subgenres and hybrid structures from music samples.

## 6 Conclusion

MGC has evolved as an important research field in the modern world with the increase in music systems and libraries. The MGC continues to be challenging due to intricately intertwined temporal, spectral, and descriptor factors. Conventional classification systems typically rely on spectrogram modality or handcrafted feature sets but do not allow for a comprehensive view of the musical structure. This study proposes a novel dual-track architecture to leverage both spectrogram-based and low-level descriptor feature representations for effective MGC. The architecture adopts the EAFRAN and the RCAEN networks that permit a balance of contextual feature extraction and statistical signal characterization. The EAFRAN leverages complex time-frequency dependencies by utilizing modules of HARF and AICA, while RCAEN generates a refined feature space using acoustic and perceptual characteristics through residual and attention mechanisms. The architecture produces refined features that contain both the fine-grained details of spectro-temporal dynamics as well as statistical descriptors that will enhance the overall understanding of musical structures. Results from evaluation of the architecture, using the GTZAN dataset, present improved performance results with an accuracy of 98.47 ± 0.6%. This performance highlights the efficacy of the novel architectural design of the proposed system to combine statistical acoustic modeling with deep attention-based learning.

## Data Availability

Publicly available datasets were analyzed in this study. This data can be found here: https://www.kaggle.com/datasets/andradaolteanu/gtzan-dataset-music-genre-classification.
